# Critically ill patients with necrotizing soft tissue infections in the Caribbean area: unsupervised analysis of a retrospective cohort (2014–2023) with identification of factors associated with mortality

**DOI:** 10.1186/s13613-025-01488-2

**Published:** 2025-06-02

**Authors:** Jean-David Pommier, Benoît Tressieres, Pascal Blanchet, Frederic Desmoulins, Pascale Piednoir, Nejla Aissa, Frederic Martino, Marc Valette, Alexandre Demoule, Sebastien Breurec, Laurent Camous

**Affiliations:** 1 Réanimation Médicale et Chirurgicale, CHU de Guadeloupe, 97139 Les Abymes, Guadeloupe, France; 2 Centre d’Investigation Clinique Antilles-Guyane, Inserm CIC 1424, CHU de Guadeloupe, 97139 Les Abymes, Guadeloupe, France; 3 Service d’Urologie, CHU de Guadeloupe, 97139 Les Abymes, Guadeloupe, France; 4 Service de Chirurgie Orthopédique, CHU de Guadeloupe, 97139 Les Abymes, Guadeloupe, France; 5 Laboratoire de Microbiologie, CHU de Guadeloupe, Chemin Chauvel, 97139 Les Abymes, Guadeloupe, France; 6 INSERM, BIGFR, F-75015 Paris, Université de Paris and Université des Antilles, Paris, France; 7 Service de Médecine Intensive et Réanimation (Département R3S), 75013 Paris Groupe Hospitalier Universitaire, Assistance Publique, Hôpitaux de Paris, Sorbonne Université, Site Pitié-Salpêtrière, Paris, France; 8 INSERM, UMRS1158 Neurophysiologie Respiratoire Expérimentale Et Clinique, 75005 Paris, France; 9 Département de Pathogenèse et Contrôle des Infections Chroniques et Émergentes, Université de Montpellier, INSERM, Etablissement Français du Sang, Montpellier, France; 10 Groupe Microbiologie Clinique, Institut Pasteur de Guadeloupe, Les Abymes, Guadeloupe, France; 11 Service de Réanimation, Centre Hospitalier Universitaire de Guadeloupe, Chemin Chauvel, 97139 Les Abymes, Guadeloupe, France

**Keywords:** Intensive care unit, Septic shock, Necrotizing soft tissue infection, Fasciitis, Multiple-organ failure

## Abstract

**Background:**

Scarce epidemiological data are available regarding necrotizing soft tissue infections (NSTIs) in tropical areas. Here we aimed to describe the clinical and biological features, and outcomes, of critically ill patients with NSTIs admitted to an intensive care unit (ICU) in a tropical setting. Furthermore, we analyzed these findings to identify distinct clinical phenotypes and explore their associations with patient outcomes.

**Methods:**

This retrospective observational study included all patients with NSTIs admitted to the ICU of the University Hospital of Guadeloupe between January 2014 and December 2023. Subgroups of patients having similar clinical profiles were identified through unsupervised clustering (factor analysis for mixed data, and hierarchical clustering on principal components). Univariate and multivariate analyses identified factors associated with 90-day mortality.

**Results:**

During the study period, 91 NSTI patients were admitted to the ICU. The median Simplified Acute Physiology Score (SAPS) II was 45 [IQR 40–66], and the median time between hospital admission and first surgical debridement was 8 h [IQR 6–10 h]. While in the ICU, 65% of patients were mechanically ventilated, 75% experienced shock, and 34% underwent renal replacement therapy. The 90-day mortality rate was 32%. Unsupervised clustering revealed three clusters—mild NSTI (*n* = 23, 25%), severe NSTI (*n* = 49, 54%), and fulminant NSTI (*n* = 19, 21%)—which were associated with different ICU courses and outcomes. Subcutaneous emphysema and sepsis-associated encephalopathy were key components influencing cluster identification. Multivariate analysis revealed that mortality was associated with SAPS II, subcutaneous emphysema, >8 h between hospital admission and first surgery, and immunocompromised status.

**Conclusion:**

Unsupervised analysis of critically ill patients with NSTIs in tropical settings revealed three distinct patient clusters that exhibited unique phenotypic characteristics and clinical outcomes. Upon hospital admission, patients with NSTIs should be carefully screened for sepsis-associated encephalopathy, subcutaneous emphysema, and thrombopenia. The present exploratory results must be confirmed in larger multicentric cohorts.

**Supplementary Information:**

The online version contains supplementary material available at 10.1186/s13613-025-01488-2.

## Background

Necrotizing soft tissue infections (NSTIs) are severe diseases characterized by extensive tissue necrosis [1–3], displaying pathological invasion of the skin, sub-cutaneous tissue, and fascia [3]. Although any body part can be affected, large epidemiological studies indicate that the lower limbs are the predominant location [4–6]. Treatment frequently requires intensive care unit (ICU) admission [2], and prolonged stays in the ICU and hospital [7], due to clinical severity, the complexity of nursing care, and the need for multiple surgical procedures.

NSTI cCase management entails a multimodal approach with close coordination of urgent interventions, including administration of broad-spectrum antibiotics, prompt surgical intervention for tissue debridement, and ICU supportive care [8]. Initial NSTI diagnosis and identification of high-risk patients are challenging [8, 9], and are hampered by two major vulnerabilities: the potential for misdiagnosis within the critical first hours after hospital admission [9], and the tendency to initially underestimate the severity of the patient’s condition [3]. Limited predictive tools are available for identifying high-risk patients, leaving physicians to rely on the usual severity scores [10–12]. Some researchers have proposed use of the Laboratory Risk Indicator for Necrotizing Fasciitis (LRINEC) score [8]; however, this score is primarily used to distinguish NSTI from other skin infections, and has limited utility beyond this scope, and its association with outcome among ICU patients remains controversial [13]. There remains a need for simple clinical and biological markers that can be tested upon hospital or ICU admission, to help physicians promptly identify patients with a poor prognosis, and to facilitate timely discussions regarding potential surgical or medical interventions [14]. Notably, only scarce epidemiological data are available regarding NSTI in tropical areas, and previous clinical data and bacteriological descriptions of NSTI from large series in tropical areas have yielded heterogenous results [5, 15, 16].

In the present study, we aimed to describe NSTI characteristics and management among patients admitted to the ICU in a tropical area, over a 10-year period. We performed an unsupervised analysis based on common clinical and biological parameters recorded at ICU admission, to identify distinct clinical phenotypes and to examine potential associations between these phenotypes and patient outcomes. In this report, we describe the outcomes, and the factors associated with 90-day mortality.

## Methods

### Study design

This retrospective study was conducted in the ICU of Guadeloupe University Hospital (GUH), which has a capacity of 38 beds, from January 2014 to December 2023. Guadeloupe is an overseas French archipelago in the West Indies, characterized by a tropical climate, and having 450,000 inhabitants. This study follows the Strengthening the Reporting of Observational Studies in Epidemiology (STROBE) statement, and was performed in accordance with the principles of the Helsinki Declaration of 1975. The protocol was approved by the local ethics committee (number A126-20/02/2024), which waived consent for retrospective anonymous data collection, in accordance with French Law.

### Patient selection

Cases were identified from the computerized medical records and laboratory microbiology databases of the GUH. Our analysis included all consecutive adult patients (>18 years old) who were admitted to the ICU with NSTI. Each NSTI diagnosis was verified by macroscopic tissue examination during the initial surgery, which is considered the gold standard procedure [10, 17].

### Data collection and syndrome definitions

Age, sex, and underlying comorbidities were recorded at ICU admission. Immunocompromised status was defined according to criteria established in a recent ICU cohort study [10], which included malignancy under chemotherapy, liver cirrhosis, and/or immunosuppressive treatment. Upon hospital admission, clinical characteristics were recorded, including body temperature; phlyctena; subcutaneous emphysema; and sepsis-associated encephalopathy [18, 19], defined as a persistent altered state of consciousness with a Glasgow coma scale of <15, after excluding alternative diagnosis. Subcutaneous emphysema was clinically and/or radiologically diagnosed. Notably, all NSTI patients underwent CT at our center.

Shock was defined as vasopressor requirement, and was evaluated at ICU admission and daily until discharge. We recorded the time between the onset of symptoms and hospital admission, and the time between hospital admission and surgery. Severity at ICU admission was assessed using the Simplified Acute Physiology Score (SAPS) II. Biological data were collected upon hospital admission, including the levels of lactate, creatinine, creatine phosphokinase (CPK), lactate dehydrogenase (LDH), and C-reactive protein (CRP), as well as leukocyte and platelet counts. Information was recorded about the number of surgical reinterventions, and regarding amputation for the subpopulation of patients who were eligible for the procedure, i.e. those with limb NSTIs located under the upper third of the arm or leg. Empirical antibiotic therapy was classified as inappropriate if the treatment regimens used within the first 24 h lacked in vitro activity against the identified bacteria. Additionally, for each patient, we documented any advanced life support therapy during the ICU stay, e.g. invasive mechanical ventilation lasting over 24 h, catecholamine use, and renal replacement therapy [20]. Reintervention was defined as a surgical procedure performed in the operating room by the surgeon, and comprised removal of bandages, thorough examination, and debridement depending on clinical data. Finally, 90-day mortality and length of ICU stay were recorded.

### Microbiological data

Bacteriological results from analyses of blood cultures and extensive soft tissue sample cultures were retrieved from the laboratory informatics system. Depending on the study period, clinical isolates were identified using the Api 20E system (bioMérieux, Marcy l’Étoile, France) or matrix-assisted laser desorption ionization time-of-flight mass spectrometry (MALDI-TOF MS) (VITEK^®^ MS system; bioMérieux, Marcy l’Etoile, France), following the manufacturer’s recommendations. Antibiotic susceptibility was evaluated using the disk diffusion method on Mueller–Hinton agar (bioMérieux, Marcy l’Étoile, France), following the guidelines of the Antibiogram Committee of the French Society of Microbiology-European Committee on Antimicrobial Susceptibility Testing (CA-SFMEUCAST). Identified Gram-positive cocci with known toxin production included Group A *Streptococcu*s and *Staphylococcus aureus* bacteria.

### Statistical analysis

Analyses were performed using R (version 4.3.0) [21]. Continuous variables are reported as median [IQR; 25th–75th quartiles], and categorical data as percentages. Subgroups of patients who exhibited similar clinical and biological profiles were identified through unsupervised clustering. First, we performed factor analysis on mixed data (FAMD), which is a factorial method used to explore associations between qualitative or quantitative variables, and to reduce the data dimensionality of a data set into non-correlated dimensions (principal components), which are linear combinations of the initial variables. Next, we performed hierarchical clustering on principal components (HCPC) constructed from the results of FAMD. HCPC is an algorithm that uses calculated principal components to partition individuals into homogeneous groups, such that the within-group similarities are large compared to the between-group similarities. The data set used for this clustering analysis included anterior wounds, shock, subcutaneous emphysema, sepsis-associated encephalopathy, Gram-negative bacilli, lactate, and platelets at ICU admission. Missing data were imputed using the missMDA package [22]. FAMD and HCPC were performed using the FactoMineR package [22].

Clusters were compared using the Kruskal–Wallis test for continuous variables, and the chi-squared or Fisher’s exact test for categorical variables. Pairwise comparisons were performed using the Dunn post-hoc test for continuous variables, and Fisher’s exact test for categorical variables, with Benjamini–Hocheberg correction. A Cox model was used to identify factors associated with mortality within 90 days of ICU admission. Variables were screened using backward stepwise Cox regression. Only variables assessed within 24 h after admission were included in the model. If a composite score (i.e. SAPS II) was included in the multivariable model, the individual components of that score were not included in the final model. Values determined to be significant (*p* < 0.05) in univariate analysis were included in multivariate analysis. We generated separate 90-day survival curves for relevant parameters, and then compared these curves using log-rank tests. For all tests, results were considered significant at 5% (*p* < 0.05). To assess the predictive performance of clusters (categorized by terciles) and SAPS II on death, we calculated their sensitivity, specificity, positive predictive value (PPV), and negative predictive value (NPV) to determine the relative accuracy and reliability of each predictor for identifying mortality risk.

## Results

### Patients’ characteristics and ICU management

During the study period, 91 patients with NSTIs were admitted to the ICU of GUH. Table 1 presents the clinical and biological features of this population. The patients were mostly male (*n* = 67, 74%). At ICU admission, the median age was 59 years [50–68 years], and median SAPS II was 46 [40–66]. Underlying conditions included diabetes in 58% (*n* = 53), cancer in 11% (*n* = 10), and cirrhosis in 11% (*n* = 10). Within the cohort, 9% (*n* = 8) were receiving immunosuppressive therapy, and 27% (*n* = 25) were immunocompromised. Additionally, 14% of the patients (*n* = 13) reported non-steroidal anti-inflammatory treatment before hospital admission.

**Table 1:** Clinical and biological characteristics of the three patient clusters

	All *N* = 91	Cluster 1 *N* = 23	Cluster 2 *N* = 49	Cluster 3 *N* = 19	*p*
Demographics					
Female, *n* (%)	24 (26)	6 (26)	15 (31)	3 (16)	0.461
Age (years)	59 (50–68)	53 (43–63)	62 (50–68)	62 (58–73)	0.074
Comorbidities					
Diabetes, *n* (%)	53 (58)	14 (61)	29 (59)	10 (53)	0.848
Body mass index (kg/m^2^)	30 (24–34)	28 (21–30)	32 (25–35)	27 (22–34)	0.027
Malignancy, *n* (%)	10 (11)	2 (9)	4 (8)	4 (21)	0.348
Cirrhosis, *n* (%)	10 (11)	1 (4)	4 (8)	5 (26)	0.081
Immunosuppressive treatment, *n* (%)	8 (9)	1 (4)	6 (12)	1 (5)	0.612
Immunocompromised status, *n* (%)	25 (27)	4 (17)	12 (25)	9 (47)	0.076
Non-steroidal anti-inflammatory treatment before admission, *n* (%)	13 (14)	5 (22)	7 (14)	1 (5)	0.339
Localization					0.028
Cervico-facial	4 (4)	3 (13)	1 (2)	0 (0)	
Limb	59 (65)	13 (57)	29 (59)	17 (90)	
Abdo-perineal region	28 (31)	7 (30)	19 (39)	2 (11)	
Time between					
Symptom onset and surgery (days)	7 (5–8)	7 (5–9)	7 (5–8)	6 (5–7)	0.366
Hospital admission and surgery (h)	8 (6–10)	7 (5–8)	8 (6–10)	12 (9–15)	<0.001
Clinical features and ICU management					
Temperature (°C)	38 (36–39)	39 (37–39)	38 (36–39)	38 (35–39)	0.561
Subcutaneous emphysema, *n* (%)	30 (33)	2 (9)	14 (29)	14 (74)	<0.001
Phlyctena, *n* (%)	37 (41)	7 (30)	17 (35)	13 (68)	0.020
Sepsis-associated encephalopathy, *n* (%)	22 (24)	1 (4)	6 (12)	15 (79)	<0.001
SAPS 2 score	46 (40–66)	35 (24–40)	46 (40–60)	79 (62–102)	<0.001
Shock, *n* (%)	68 (75)	0 (0)	49 (100)	19 (100)	<0.001
Mechanical ventilation, *n* (%)	59 (65)	4 (17)	37 (76)	18 (95)	<0.001
Renal replacement therapy, *n* (%)	31 (34)	2 (9)	17 (35)	12 (63)	0.001
Surgical reintervention (*n*)	5 (3–6)	4 (3–5)	5 (3–7)	2 (0–3)	0.001
Amputation, *n* (%)	17 (19)	1 (4)	11 (22)	5 (26)	0.104
Amputation in patients eligible for the procedure, *n* (%)	17 (34)	1 (8)	11 (48)	5 (33)	0.043
Mechanical ventilation duration (days)	3 (2–8)	5 (1–7)	5 (2–10)	3 (1–5)	0.149
Dialysis duration (days)	5 (5–13)	4 (4–4)	5 (5–13)	7 (5–15)	0.304
Biological data					
Lactate (mmol/L)	2 (1–5)	1 (1–2)	2 (1–4)	7 (5–9)	<0.001
Leukocytes (G/L)	16.5 (9.9–24.5)	17.7 (12.1–22.1)	19 (11–26)	10 (3–15)	0.013
Thrombocytopenia (<50,000/mm^3^), *n* (%)	31 (34)	3 (13)	16 (33)	12 (63)	0.003
Creatinine (µmol/L)	167 (82–292)	90 (62–242)	183 (98–310)	209 (150–289)	0.083
CPK (UI/L)	350 (110–1000)	200 (110–685)	300 (100–1000)	1450 (700–9250)	<0.001
LDH (UI/L)	200 (200–390)	200 (150–288)	200 (200–354)	700 (314–934)	0.003
CRP (mg/L)	314 (224–434)	316 (222–434)	331 (225–434)	280 (233–360)	0.562
Microbiological data					
Unappropriated empirical therapy, *n* (%)	20 (22)	5 (22)	9 (18)	7 (37)	0.252
Positive blood culture, *n* (%)	16 (18)	1 (4)	9 (18)	6 (32)	0.078
Positive soft tissue culture, *n* (%)	84 (92)	19 (83)	46 (94)	19 (100)	0.097
Polymicrobial culture, *n* (%)	24 (26)	6 (26)	17 (35)	1 (5)	0.047
Gram-positive cocci producing bacterial toxins, *n* (%)	32 (35)	8 (35)	19 (39)	5 (26)	0.627
Gram-negative bacilli, *n* (%)	45 (49)	10 (44)	24 (49)	11 (58)	0.646
Outcome					
90-day mortality	29 (32)	2 (9)	14 (29)	13 (68)	<0.001
ICU length of stay	7 (3–15)	4 (2–5)	9 (5–15)	3 (2–16)	<0.001

Results are presented as median (25th–75th quartiles) for continuous variables, and *n* (percentage) for categorical variables

SAPS 2, simplified acute physiology score; Immunosuppressive status, cirrhosis or immunosuppressive treatment or malignancy; CPK, creatine phosphokinase; LDH, lactate dehydrogenase; CRP, C-reactive protein

The median duration of symptoms before surgery was 7 days [5–8 days]. At hospital admission, 24% of patients (*n* = 22) exhibited Sepsis-associated encephalopathy, among whom 30% (*n* = 5) exhibited coma, and 70% (*n* = 17) delirium. NSTI involved the limbs in 65% of patients, abdo-perineal region in 31%, and cervico-facial region in the remaining 4%. The median interval between hospital admission and surgery was 8 h [6–10 h]. Within 24 h of ICU admission, 65% (*n* = 59) were receiving mechanical ventilation, and 75% (*n* = 68) exhibited shock. During their ICU course, 34% of patients (*n* = 31) required renal replacement therapy, including 2 patients who were receiving chronic renal replacement therapy before ICU admission.

### Microbiological features and treatment

**Fig. 1:**
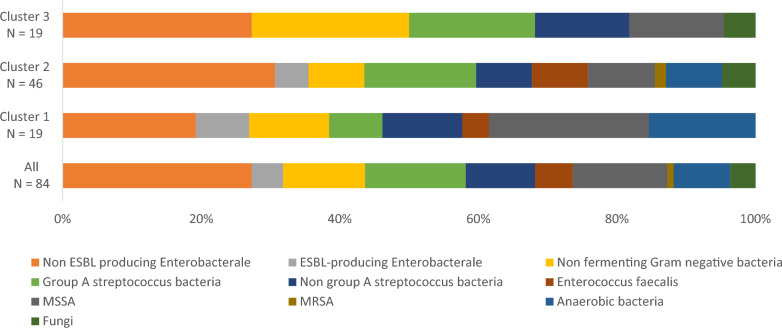
Bacteria and fungi identified from soft tissues samples or blood cultures from all patients with necrotizing soft tissue infections who had at least one identified microorganism (*N* = 84/91), and according to cluster: cluster 1 (*N* = 19/23), cluster 2 (*N* = 46/49), and cluster 3 (*N* = 19/19)

Figure 1 and Supplementary Table S1 present the predominant bacteria isolated from soft tissue samples and blood cultures. Surgical skin and soft tissue samples cultures were positive for 91% of patients (*n* = 84), while blood cultures were positive for 18% (*n* = 16). Monomicrobial NSTIs were detected in 42% (*n* = 38) of cases. Gram-negative bacilli were identified in 49% (*n* = 45), and toxin-producing Gram-positive cocci in 35% (*n* = 32). Pathogens related to NSTI Class 3 [1] were isolated in 5% of cases (*n* = 5). Multi-drug-resistant bacteria (including extended-spectrum beta-lactamase-producing Gram-negative bacteria) were detected in 5% of the cohort (*n* = 5), and 66% of these cases (*n* = 3 of 5) received inappropriate first-line antibiotherapy.

During their ICU stay, patients required a median of 5 reinterventions [3–6 reinterventions], and 19% (*n* = 17) underwent limb amputation. No patient had a nosocomial NSTI.

### Cluster analysis

Supplementary Fig. S1 presents the results of multiple component analysis (MCA) of our cohort. Cluster analysis revealed three distinct phenotypes. Table 1 presents the patients’ characteristics and ICU management according to cluster, showing no significant between-clusters difference in age or the time from symptoms onset to hospital admission. Cluster 1 (*n* = 23, 25%) was characterized by the least severe phenotype (“mild NSTI”). Patients in this group rarely exhibited sepsis-associated encephalopathy or subcutaneous emphysema at ICU admission (4% and 9%, respectively). Throughout their ICU course, patients in cluster 1 did not require vasopressor support, and had the lowest rates of mechanical ventilation (17%) and renal replacement therapy (9%). Compared to the two other clusters, cluster 1 exhibited a significantly lower 90-day mortality rate (9%) (*p* < 0.05).

Cluster 2 or “severe NSTI” (54%, *n* = 49) was the most prevalent phenotype. This group exhibited a median SAPS II of 46 [IQR 40–64]. Among patients in cluster 2, 76% (*n* = 37/49) required mechanical ventilation, 100% exhibited shock, and 35% required renal replacement therapy. Limb amputation was performed in 22%. Compared to those in cluster 1, patients in cluster 2 more frequently exhibited subcutaneous emphysema (26% vs. 9%, *p* < 0.001) and required vasopressors (100% vs. 0%, *p* < 0.001). Cluster 2 exhibited a 90-day mortality rate of 26%.

Cluster 3 or “fulminant NSTI” (*n* = 19, 21%) included the most severe cases. Compared to those in clusters 1 and 2, the patients in cluster 3 exhibited the highest median SAPS II (79 [IQR 62–102]) (*p* < 0.001). Cluster 3 experienced the poorest hospital outcomes, with a 90-day mortality rate of 68% (*p* < 0.001). In cluster 3, the predominantly NTSI localization was in the limbs, which differed from clusters 1 and 2 (*p* = 0.028). At hospital admission, patients in cluster 3 exhibited higher rates of sub-cutaneous emphysema (74%) and sepsis-associated encephalopathy (79%), as well as lower platelet counts and higher lactate levels. Compared to other clusters, cluster 3 exhibited a higher prevalence of immunocompromised status, but this difference was not statistically significant. The most commonly identified bacteria did not differ among the clusters.

### Outcome and factors associated with mortality

The overall in-hospital mortality rate was 32% (*n* = 29). Notably, among the deceased patients, 50% (*n* = 15) died of refractory shock due to NSTI, and the median time to death was 5 days [IQR 2–9], despite appropriate antibiotherapy and repeated surgical procedures. Among the other deceased patients, death occurred secondary to ICU complications: 5 after withdrawal of care procedure, 8 due to refractory nosocomial sepsis, and 1 due to hemorrhage. Table 2 shows factors associated with in-hospital mortality according to univariate analysis. Subsequent multivariate analysis revealed that in-hospital mortality was associated with SAPS II (HR 1.04, 95% CI [1.02–1.05]), >8 h between hospital admission and surgery (HR 4.41, 95% CI [1.23–15.83]), immunocompromised status (HR 2.74, 95% CI [1.24–6.05]), and the presence of subcutaneous emphysema at first clinical examination (HR 3.87, 95% CI [1.72–8.74]).

**Table 2:** Patients’ characteristics and outcomes according to 90-day survival

	Death within 90-days (*n* = 29)	90-day survivor (*n* = 62)	Univariate analysis		Multivariate analysis	
			HR (95% CI)	*p*	HR (95% CI)	*p*
Demographics						
Female, *n* (%)	5 (17)	19 (31)	0.51 (0.19–1.33)	0.167		
Age (years)	59 (54–67)	60 (48–68)	1.01 (0.99–1.04)	0.331		
Comorbidities						
Diabetes, *n* (%)	17 (59)	36 (58)	1.01 (0.48–2.12)	0.975		
Body mass index (kg/m^2^)	30 (23–33)	30 (24–34)	0.99 (0.95–1.04)	>0.999		
Malignancy, *n* (%)	6 (21)	4 (7)	2.83 (1.15–6.97)	0.023		
Cirrhosis, *n* (%)	5 (17)	5 (8)	2.04 (0.78–5.34)	0.149		
Immunosuppressive treatment, *n* (%)	4 (14)	4 (7)	1.85 (0.64–5.31)	0.255		
Immunocompromised status, *n* (%)	13 (45)	12 (19)	2.73 (1.31–5.68)	0.007	2.74 (1.24–6.05)	0.013
Time between						
Symptom onset and surgery (days)	6 (5–7)	7 (5–9)	0.97 (0.89–1.05)	0.434		
Hospital admission and surgery over 8 h, *n* (%)	26 (90)	23 (39)	9.55 (2.88–31.6)	<0.001	4.41 (1.23–15.83)	0.023
Clinical features and ICU management						
Temperature (°C)	37 (35–39)	38 (37–39)	0.86 (0.68–1.08)	0.187		
Subcutaneous emphysema, *n* (%)	20 (69)	10 (16)	6.94 (3.15–15.3)	<0.001	3.87 (1.72–8.74)	0.001
Phlyctena, *n* (%)	15 (52)	22 (36)	1.79 (0.86–3.70)	0.118		
SAPS 2 score	74 (55–95)	40 (35–55)	1.04 (1.03–1.06)	<0.001	1.04 (1.02–1.05)	<0.001
Sepsis-associated encephalopathy, *n* (%)	13 (45)	9 (15)	3.55 (1.70–7.40)	<0.001		
Shock, *n* (%)	27 (93)	41 (66)	5.42 (1.29–22.8)	0.021		
Mechanical ventilation, *n* (%)	24 (83)	35 (57)	3.09 (1.18–8.10)	0.022		
Renal replacement therapy, *n* (%)	15 (52)	16 (26)	2.50 (1.20–5.18)	0.014		
Surgical reintervention (*n*)	5 (1–8)	4 (3–6)	1.02 (0.90–1.16)	0.769		
Amputation, *n* (%)	3 (10)	14 (23)	0.47 (0.14–1.56)	0.218		
Amputation in patients eligible for the procedure, *n* (%)	3/19 (14)	14/31 (45)	4.26 (0.94–27.50)	0.06		
Biological data						
Lactate (mmol/L)	5 (2–7)	2 (1–3)	1.22 (1.12–1.33)	<0.001		
Leukocytes (G/L)	13 (6–25)	17 (12–24)	0.98 (0.94–1.01)	0.204		
Thrombocytopenia (<150,000/mm^3^), *n* (%)	16 (55)	15 (24)	3.10 (1.49–6.47)	0.002		
Creatinine (µmol/L)	273 (164–378)	117 (71–247)	1.00 (1.00–1.00)	0.020		
CPK (UI/L)	850 (178–1425)	300 (103–1000)	1.00 (1.00–1.00)	0.728		
LDH (UI/L)	307 (188–700)	200 (200–312)	1.00 (1.00–1.00)	0.005		
CRP (mg/L)	280 (213–413)	318 (228–437)	1.00 (1.00–1.00)	0.278		
Microbiological data						
Toxic Gram-positive cocci, *n* (%)	13 (45)	21 (34)	1.58 (0.57–4.44)	0.350		
Gram-negative bacilli, *n* (%)	15 (52)	30 (48)	1.19 (0.57–2.46)	0.647		
Cluster						
1. Mild NSTI group, *n* (%)	2 (7)	21 (34)	Ref			
2. Severe NSTI group, *n* (%)	14 (48)	35 (57)	3.43 (0.78–15.1)	0.103		
3. Fulminant NSTI group, *n* (%)	13 (45)	6 (10)	15.1 (3.39–67.2)	<0.001		

Results are presented as median (25th–75th quartiles) for continuous variables, and *n* (percentage) for categorical variables

HR, hazard ratio; 95% CI, 95% confidence interval; SAPS 2, simplified acute physiology score; Immunosuppressive status, cirrhosis or immunosuppressive treatment or malignancy; CPK, creatine phosphokinase; LDH, lactate dehydrogenase; CRP, C-reactive protein

**Fig. 2:**
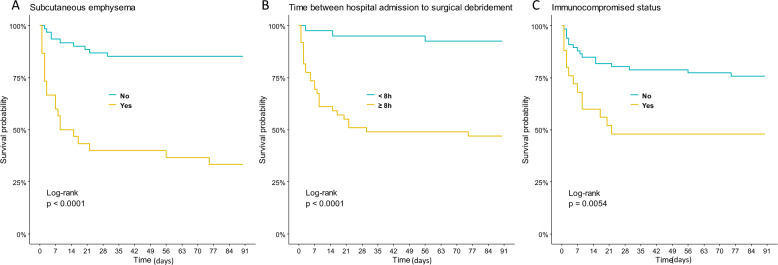
Cumulative survival rates. Kaplan–Meier survival curves of patients admitted to the intensive care unit with necrotizing soft tissue infections, stratified according to the presence or absence of subcutaneous emphysema (**A**), time from hospital admission to surgical debridement (within or over 8 h) (**B**), and immunocompromised status (**C**)

Supplementary Table S2 presents the clinical and biological features of immunocompromised patients, and Supplementary Table S3 provides details regarding NSTI localization. Notably, we found no major differences between immunocompromised and non-immunocompromised patients, with the exceptions of hospital outcome and time between hospital admission and surgery. Specifically, surgery was performed more than 8 h after admission among 75% (*n* = 31) of immunocompromised patients, compared to 48% (*n* = 18) of non-immunocompromised patients (*p* = 0.022). Figure 2 illustrates the cumulative survival rates according to the presence of subcutaneous emphysema, the delay between hospital admission and surgery, and immunocompromised status. Supplementary Table S4 and Fig. S1 present the performance of SAPS II (categorized by terciles) versus cluster for predicting 90-day mortality. Cluster 3 had a higher specificity of 0.90 (0.80, 0.96) when compared to SAPS II, but a lower sensitivity of 0.45 (0.26, 0.64).

## Discussion

Here we report a large cohort of patients with NSTIs, who were admitted to an ICU in a tropical area. Compared to previous reports of cohorts from temperate areas, the NSTIs in our cohort were characterized by a shorter interval between the onset of symptoms and hospital admission. This difference may reflect unique factors related to the climatic conditions in tropical areas [5, 23]. The mortality rate in our study (32%) was consistent with the value by the SAPS II score, and is comparable to the rates reported in large studies from temperate areas [5, 6, 8–12, 24, 25]. In one multicenter international cohort [7], the 90-day mortality rate was 18%, which is substantially lower than in our study. However, the populations differed in several ways—with our cohort showing a higher rate of comorbidities and higher SAPS II score at baseline, as well as higher rates of renal replacement therapy and septic shock during the ICU course.

Remarkably, our present study was the first to identify distinct clinical phenotypes upon hospital admission, which correlate with 90-day survival outcomes. We performed MCA within our ICU cohort, which revealed three distinct clinical phenotypes of NSTI cases admitted to the ICU: cluster 1 (mild NSTIs), cluster 2 (severe NSTIs), and cluster 3 (fulminant NSTIs). These classifications, which are exploratory, could provide significant insights into the diverse spectrum of NSTIs, potentially enhancing both prognostic evaluation and therapeutic interventions. Cluster 1 exhibited a better prognosis at day 90, with patients less frequently experiencing severe organ failure, and requiring fewer mechanical replacement therapies while in the ICU. Cluster 2 was the predominant group, and mainly included critically ill NSTI patients. Cluster 3 comprised the most severe cases, which were characterized by a distinct clinical and biological phenotype upon hospital admission. Compared to clusters 1 and 2, cluster 3 exhibited higher frequencies of sepsis-associated encephalopathy and subcutaneous emphysema, a lower platelet count, higher initial lactatemia, and higher CPK level. These factors were key markers for cluster identification, and could thus serve as crucial bedside indicators for physicians. Interestingly, all NSTI patients showed elevated CRP levels, which did not significantly differ between clusters, and were not significantly associated with patient outcome.

The underlying comorbidities in cluster 3 were similar to those in clusters 1 and 2; however, there was a tendency towards a higher rate of immunocompromised patients in cluster 3 (*p* = 0.07), which may be attributed to limited statistical power. Moreover, the time from symptom onset to hospital admission among patients in cluster 3 was comparable to that among patients in clusters 1 and 2. This finding suggests that the cluster 3 phenotype exhibited rapid clinical progression of NSTI, rather than being late-stage disease at presentation. Notably, the time from hospital admission to surgery was significantly longer in cluster 3, compared to in the two other clusters, which might be related to the higher prevalence of immunocompromised patients (47%) in cluster 3. Indeed, immunocompromised patients are often misdiagnosed due to the initial scarcity of clinical signs in this population, which reportedly increases the delay between emergency room admission and surgery [26]. However, this was probably not the only determinant, since immunocompromised patients in clusters 1 and 2 displayed different clinical phenotypes. Hence, we propose that the “fulminant NSTI” phenotype represents a subpopulation characterized by an early and severe clinical course, which is likely influenced by multifactorial determinants, including host, bacterial, and environmental factors [27].

There remains a need for additional studies, in both tropical and non-tropical settings, to validate our exploratory findings. Considering the grim prognosis of the subpopulation with fulminant NSTIs, future studies should assess the impact of additional therapeutic interventions. Authors have proposed that early amputation may be beneficial for severe NSTIs [28, 29], although there is no clear evidence of an impact on prognosis (as in our present study). Notably, the available studies are retrospective with many biases, and prospective studies could be considered among highly selected patients.

Previous efforts to identify factors associated with in-ICU mortality among NSTIs patients have yielded heterogeneous results [2, 4, 6–8, 11, 12]. However, our present data support a growing consensus regarding the importance of two key predictors of NSTI outcomes [4, 6, 7, 10–12]: the time between hospital admission and surgery; and the severity at ICU admission, as reflected by SAPS II score. Interestingly, our multivariate analysis also revealed that immunocompromised status was independently associated with death. While previous studies have shown adverse outcomes of NSTI in immunocompromised individuals [10, 26], immunocompromised status has not been reported to be independently related to death in prior cohorts [5–7, 10, 11]. In our cohort, 22% of patients were immunocompromised, which is similar to the proportion observed in a recent French multicenter cohort [10].

To explain why immunocompromised status was independently related to death in our cohort from a tropical region, several plausible hypotheses can be considered. First, in tropical settings, specific subpopulations exhibit a notably worse prognosis for skin infection following marine-related wounds [15], particularly due to rapid and severe disease progression. Our study did not specifically examine exposure to marine water; however, pathogens commonly associated with marine wound infections (e.g., *Vibrio vulnificus* and *Aeromonas* spp. [15, 16]) were rarely isolated in our cohort (5% of cases), potentially indicating limited exposure. Second, climate factors, including temperature and hygrometry, may influence the observed features of skin infection. For example, warmer temperatures can accelerate bacterial growth, especially for bacteria of clinical interest, and enhance the virulence of pathogens [30]. Moreover, high humidity may favor prolonged skin moisture, which could contribute to skin barrier disruption, thereby facilitating pathogen entry. It is possible that these elements, together with patients’ immunocompromised status, may have contributed to the observed higher mortality. However, larger cohort studies in tropical regions are needed to validate these hypotheses.

Subcutaneous emphysema is a classical sign of NSTIs, and was present in 33% of our patients, which is at the higher range of previously reported values (10–30%) from cohorts in temperate regions [3, 7, 10]. Traditionally linked with later disease stages [1], subcutaneous emphysema has not, to our knowledge, been previously shown to have prognostic significance in NSTIs. We hypothesize that NSTIs in tropical areas may exhibit two distinctive features—accelerated infectious progression, and early emergence of subcutaneous emphysema—possibly influenced by climate and hygrometry factors [23, 27].

The comorbidities among our patients align with those classically described in previous studies [10, 14], with diabetes and immunosuppression identified as the main predisposing factors. On the other hand, our bacteriological findings included a predominance of Gram-negative bacilli (50%), and strongly contrast with those classically reported in temperate areas [5], where Gram-positive cocci have been predominant, even if recent data differ [10]. Observational data suggest that elevated temperatures and high hygrometry in tropical settings can shift the predominant isolated bacteria in NSTIs toward Gram-negative bacteria [23], while Gram-positive cocci are more prevalent under cooler and drier conditions.

We acknowledge several limitations in the present work. First, the retrospective design and monocentric nature may have introduced selection bias. For example, the NSTI severity was higher in our cohort compared to other studies [7], which may limit the generalizability of our findings. Second, the small sample size means that the results of our multivariate analysis must be interpreted with caution, highlighting the need for validation with larger studies in both tropical and non-tropical settings. Nonetheless, given the extensive population coverage of the GUH, and its role as a referral center for NSTIs in the region, we believe that our present data provide a representative overview of NSTI characteristics in Guadeloupe and, by extension, in tropical areas.

## Conclusion

NSTIs are severe infections, associated with high mortality and prolonged hospital course; however, their epidemiological and clinical features in tropical areas have been scarcely studied. Early diagnosis, emergency care, and prompt surgery are major determinants of patient outcome. Through our exploratory unsupervised analysis based on clinical and biological factors at hospital admission, we identified a high-risk phenotype with high ICU mortality, which is characterized by sepsis-associated encephalopathy, hyperlactatemia, and thrombopenia at hospital admission. These exploratory results must be confirmed in larger multicentric cohorts, and could lead to the development of new therapeutic strategies for subgroups of patients.

## Supplementary Information

**Additional file 1.**

## Data Availability

All data and materials are available on demand.
