# Supplementary material for: Critically ill patients with necrotizing soft tissue infections in the Caribbean area: unsupervised analysis of a retrospective cohort (2014–2023) with identification of factors associated with mortality
Source: Ann Intensive Care. 2025 Jun 2;15:77. doi: 10.1186/s13613-025-01488-2 (PMC12130383; doi:10.1186/s13613-025-01488-2)
Supplement: Supplementary file 1 — Additional file 1. [file 13613_2025_1488_MOESM1_ESM.docx]

|  | All  N=91 | Cluster 1  N=23 | Cluster 2  N=49 | Cluster 3  N=19 |
| --- | --- | --- | --- | --- |
|  |  |  |  |  |
| Patients with identified microorganism, *n (%)* | 84 (92) | 19 (83) | 46 (94) | 19 (100) |
| Plurimicrobial samples, *n (%)* | 24 (26) | 6 (26) | 17 (35) | 1 (5) |
| **Gram-negative bacilli**, *n (%)* |  |  |  |  |
| Non-ESBL-producing Enterobacterales, *n (%)* | 30 (33) | 5 (22) | 19 (41) | 6 (27) |
| *Escherichia coli* | 15 (17) | 2 (9) | 10 (20) | 3 (16) |
| *Klebsiella pneumoniae* | 7 (8) | 1 (4) | 5 (10) | 1 (5) |
| *Proteus mirabilis* | 5 (5) | 1 (4) | 3 (6) | 1 (5) |
| *Morganella morganii* | 2 (2) | 0 (0) | 2 (4) | 0 (0) |
| *Enterobacter cloacae* | 1 (1) | 1 (4) | 0 (0) | 0 (0) |
| ESBL-producing Enterobacterales, *n (%)* | 5 (5) | 2 (9) | 3 (7) | 0 (0) |
| *Enterobacter cloacae* | 1 (1) | 1 (4) | 0 (0) | 0 (0) |
| *Escherichia coli* | 1 (1) | 0 (0) | 1 (2) | 0 (0) |
| *Klebsiella pneumoniae* | 2 (2) | 1 (4) | 1 (2) | 0 (0) |
| Non-fermenting Gram-negative bacilli, *n (%)* | 13 (14) | 3 (13) | 5 (11) | 5 (13) |
| *Pseudomonas aeruginosa* | 8 (9) | 3 (13) | 4 (8) | 1 (5) |
| *Acinetobacter baumanii* | 1 (1) | 0 (0) | 1 (2) | 0 (0) |
| *Aeromonas spp.* | 4 (4) | 0 | 0 (0) | 4 (21) |
| *Vibrio vulnificus* | 1 (1) | 0 (0) | 0 (0) | 1 (5) |
| **Gram-positive cocci** |  |  |  |  |
| Group A streptococcus bacteria, *n (%)* | 16 (18) | 2 (9) | 10 (22) | 4 (18) |
| Non-group A streptococcus bacteria, *n (%)* | 11 (12) | 3 (13) | 5 (11) | 3 (13) |
| *Streptococcus agalactiae* | 2 (2) | 0 (0) | 2 (4) | 0 (0) |
| *Streptococcus anaerobius* | 1 (1) | 0 (0) | 1 (2) | 0 (0) |
| *Streptococcus angionosus* | 2 (2) | 1 (4) | 1 (2) | 0 (0) |
| *Streptococcus constellatus* | 3 (3) | 1 (4) | 2 (4) | 0 (0) |
| *Streptococcus dysgalactiae* | 1 (1) | 0 (0) | 0 (0) | 1 (5) |
| *Streptococcus F.* | 1 (1) | 1 (4) | 0 (0) | 0 (0) |
| *Streptococcus mitis* | 1 (1) | 0 (0.0%) | 0 (0) | 1 (5) |
| *Enterococcus faecalis* | 6 (7) | 1 (4) | 5 (11) | 0 (0) |
| Methicillin-sensitive *Staphylococcus aureus*, *n (%)* | 15 (16) | 6 (26) | 6 (13) | 3 (14) |
| Methicillin-resistant *Staphylococcus aureus*, *n (%)* | 1 (1) | 0 (0) | 1 (2) | 0 (0) |
| **Gram-positive bacillus** |  |  |  |  |
| *Corynebacterium spp.* | 1 (1) | 0 (0) | 0 (0) | 1 (5) |
| **Anaerobic bacteria**, *n (%)* | 9 (10) | 4 (17) | 5 (11) | 0 (0) |
| *Actinomyces spp.* | 4 (5) | 2 (8) | 2 (4) | 0 (0) |
| *Fusobacterium spp.* | 1 (1) | 1 (4) | 0 (0) | 0 (0) |
| *Bacteroides fragilis* | 1 (1) | 0 (0) | 1 (2) | 0 (0) |
| *Clostridium innocuum* | 1 (1) | 1 (4) | 0 (0) | 0 (0) |
| Other | 2 (2) | 0 (0) | 2 (4) | 0 (0) |
| **Fungi**, *n (%)* | 4 (4) | 0 (0) | 3 (7) | 0 (0) |
| *Candida albicans* | 3 (1) | 0 (0) | 3 (6) | 0 (0) |
| *Fusarium* | 1 (1) | 0 (0) | 0 (0) | 1 (5) |

**Table S1. Microorganisms identified from soft tissues samples or blood cultures from all patients with necrotizing soft tissue infections and at least one identified microorganism, stratified according to cluster.**

ESBL, extended-spectrum beta-lactamase.

**Table S2. Patients’ characteristics and outcomes according to immunocompromised status.**

|  | Patients without immunocompromised status  (n=66) | Patients with immunocompromised status  (n=25) |  |
| --- | --- | --- | --- |
|  |  |  | p |
| **Demographics** |  |  |  |
| Female, *n (%)* | 21 (32) | 3 (12) | 0.055 |
| Age, *years* | 58 (49–67) | 63 (54–72) | 0.169 |
| **Time between** |  |  |  |
| Symptom onset and surgery, *days* | 7 (5–8) | 7 (4–7) | 0.595 |
| Hospital admission and surgery over 8 hours, *n (%)* | 18 (31) | 5 (24) | 0.560 |
| **Clinical features and ICU management** |  |  |  |
| Temperature, *°C* | 38 (37–39) | 38 (35–39) | 0.103 |
| Subcutaneous emphysema, *n (%)* | 19 (29) | 11 (44) | 0.168 |
| Phlyctena, *n (%)* | 29 (44) | 8 (32) | 0.301 |
| Sepsis-associated encephalopathy, *n (%)* | 13 (20) | 9 (36) | 0.105 |
| SAPS 2 score | 44 (36–66) | 54 (44–62) | 0.146 |
| Shock, *n (%)* | 47 (71) | 21 (84) | 0.210 |
| Mechanical ventilation, *n (%)* | 41 (62) | 18 (72) | 0.378 |
| Renal replacement therapy, *n (%)* | 19 (29) | 12 (48 | 0.084 |
| Surgical reintervention, *n (%)* | 5 (3–6) | 4 (1-7) | 0.486 |
| Amputation, *n (%)* | 14 (21) | 3 (12) | 0.382 |
| Amputation in patients eligible for the procedure, n (%) | 14 (39) | 3 (21) | 0.374 |
| Mechanical ventilation duration *(days)* | 3 (2 – 8) | 3 (2 – 9) | 0.726 |
| Dialysis duration *(days)* | 8 (8 – 8) | 8 (6 – 8) | 0.979 |
| **Biological data** |  |  |  |
| Lactate, *mmol/L* | 2 (1–4) | 4 (2–7) | 0.004 |
| Leukocytes, *G/L* | 17 (11–25) | 14 (5–24) | 0.351 |
| Thrombocytopenia (< 150000/mm3), n (%) | 17 (26) | 14 (56) | 0.009 |
| Creatinine, *µmol/L* | 138 (73–286) | 209 (150–346) | 0.067 |
| CPK, *UI/L* | 300 (107–1000) | 850 (200–3805) | 0.089 |
| LDH, *UI/L* | 200 (200–353) | 292 (150–742) | 0.420 |
| CRP, *mg/L* | 340 (232–450) | 255 (160–311) | 0.006 |
| **Microbiological data** |  |  |  |
| Unappropriated empirical therapy, *n (%)* | 12 (19) | 8 (32) | 0.187 |
| Positive blood culture, *n (%)* | 9 (14) | 7 (37) | 0.052 |
| Positive soft tissue culture, *n (%)* | 59 (89) | 25 (100) | 0.183 |
| Polymicrobial culture, *n (%)* | 18 (1) | 6 (24) | 0.567 |
| Gram-positive cocci producing bacterial toxins, *n (%)* | 23 (35) | 9 (36) | 0.918 |
| Gram-negative bacilli, *n (%)* | 31 (47) | 14 (56) | 0.442 |
| **Cluster** |  |  | 0.081 |
| 1, *n (%)* | 19 (29) | 4 (16) |  |
| 2, *n (%)* | 35 (53) | 11 (44) |  |
| 3, *n (%)* | 12 (18) | 10 (40) |  |
| **Outcome** |  |  |  |
| 90-day mortality | 16 (24) | 13 (52) | 0.011 |
| ICU length of stay | 7 (3–15) | 7 (2–15) | 0.741 |
| SAPS 2, simplified acute physiology score; Immunosuppressive status, cirrhosis or immunosuppressive treatment or malignancy; CPK, creatine phosphokinase; LDH, lactate dehydrogenase; CRP, C-reactive protein. | | | |

**Table S3. Patients’ characteristics and outcomes according to necrotizing soft tissue infection localization.**

|  | Cervico-facial region  N=4 | Limbs  N=59 | Abdo-perineal region N=28 | p value |
| --- | --- | --- | --- | --- |
| **Demographics** |  |  |  |  |
| Female, *n (%)* | 0 (0) | 18 (31) | 6 (21) | 0.367 |
| Age, *years* | 44 (32–57) | 60 (50–68) | 59 (50–68) | 0.348 |
| **Comorbidities** |  |  |  |  |
| Diabetes, *n (%)* | 1 (25) | 36 (61) | 16 (57) | 0.414 |
| Malignancy, *n (%)* | 0 (0) | 7 (12) | 3 (11) | 0.999 |
| Cirrhosis, *n (%)* | 0 (0) | 6 (10) | 4 (14) | 0.826 |
| Immunosuppressive treatment, *n (%)* | 0 (0) | 8 (14) | 0 (0) | 0.097 |
| Immunocompromised status, *n (%)* | 0 (0) | 18 (31) | 7 (25) | 0.600 |
| Non-steroidal anti-inflammatory treatment before admission, *n (%)* | 3 (75) | 7 (12) | 3 (11) | 0.016 |
| **Time between** |  |  |  |  |
| Symptom onset and surgery, *days* | 1 (25) | 17 (34) | 5 (19) | 0.406 |
| Hospital admission and surgery, *hours* | 9 (8-9) | 8 (6-12) | 7 (6-9) | 0.172 |
| **Clinical features and ICU management** |  |  |  |  |
| Temperature, *°C* | 36 (36–37) | 38 (36–39) | 38 (37–39) | 0.486 |
| Subcutaneous emphysema, *n (%)* | 0 (0) | 20 (34) | 10 (36) | 0.491 |
| Phlyctena, *n (%)* | 0 (0) | 32 (54) | 5 (18) | <0.001 |
| Sepsis-associated encephalopathy, *n (%)* | 0 (0) | 18 (31) | 4 (14) | 0.144 |
| SAPS 2 score | 38 (31–40) | 46 (38–68) | 49 (40–63) | 0.146 |
| Shock, *n (%)* | 1 (25) | 46 (78) | 21 (75) | 0.078 |
| Mechanical ventilation, *n (%)* | 4 (100) | 37 (63) | 18 (64) | 0.457 |
| Renal replacement therapy, *n (%)* | 0 (0) | 25 (42) | 6 (21) | 0.052 |
| Surgical reintervention | 5 (3–5) | 4 (2–6) | 5 (4–7) | 0.201 |
| Amputation, *n (%)* | 0 (0) | 17 (29) | 0 (0) | 0.002 |
| Amputation in patients eligible for the procedure, n (%) | 0 (0) | 17 (35) | 0 (0) |  |
| Mechanical ventilation duration *(days)* | 7 (6–9) | 3 (2-8) | 2 (1-13) | 0.311 |
| Dialysis duration *(days)* | 8 (8-8) | 8 (6-8) | 8 (8-8) | 0.375 |
| **Biological data** |  |  |  |  |
| Lactate, *mmol/L* | 1 (1–1) | 3 (1–5) | 2 (1–3) | 0.008 |
| Leukocytes, *G/L* | 10 (7–14) | 18 (10–26) | 14 (11–23) | 0.480 |
| Thrombocytopenia (< 150000/mm3), n (%) | 1 (25) | 20 (34) | 10 (36) | 1.0 |
| Creatinine, *µmol/L* | 73 (57–113) | 194 (98–307) | 143 (72–239) | 0.107 |
| CPK, *UI/L* | 219 (100–529) | 900 (200–1900) | 190 (100–275) | 0.001 |
| LDH, *UI/L* | 200 (175–218) | 250 (200–429) | 200 (189–327) | 0.321 |
| CRP, *mg/L* | 122 (108–180) | 297 (233–437) | 340 (223–441) | 0.062 |
| **Microbiological data** |  |  |  |  |
| Unappropriated empirical therapy, *n (%)* | 1 (25) | 13 (22) | 6 (21) | >0.999 |
| Positive blood culture, *n (%)* | 0 (0) | 10 (17) | 6 (25) | 0.712 |
| Positive soft tissue culture, *n (%)* | 4 (100) | 54 (92) | 26 (93) | 1.0 |
| Polymicrobial culture, *n (%)* | 0 (0) | 13 (22) | 11 (39) | 0.133 |
| Gram-positive cocci producing bacterial toxins, *n (%)* | 0 (0) | 28 (48) | 4 (14) | 0.002 |
| Gram-negative bacilli, *n (%)* | 1 (25) | 26 (44) | 18 (64) | 0.154 |
| **Outcome** |  |  |  |  |
| 90-day mortality | 0 (0) | 21 (36) | 8 (29) | 0.407 |
| SAPS 2, simplified acute physiology score; Immunosuppressive status, cirrhosis or immunosuppressive treatment or malignancy; CPK, creatine phosphokinase; LDH, lactate dehydrogenase; CRP, C-reactive protein. | | | | |

**Table S4: Comparison of performance of SAPS II (categorized by terciles) and clusters on death at day-90 prediction**

| Characteristic | Death within 90-days  N = 29 | 90-day survivor  N = 62 |
| --- | --- | --- |
| SAPS II |  |  |
| < 40 | 2 (7) | 21 (34) |
| [40;60[ | 8 (28) | 30 (48) |
| ≥ 60 | 19 (66) | 11 (18) |
| cluster |  |  |
| 1 | 2 (7) | 21 (34) |
| 2 | 14 (48) | 35 (56) |
| 3 | 13 (45) | 6 (10) |

|  | SAPS II ≥ 60 | Cluster 3 |
| --- | --- | --- |
| Sensitivity | 0.66 (0.46, 0.82) | 0.45 (0.26, 0.64) |
| Specificity | 0.82 (0.70, 0.91) | 0.90 (0.80, 0.96) |
| Positive predictive value | 0.63 (0.44, 0.80) | 0.68 (0.43, 0.87) |
| Negative predictive value | 0.84 (0.72, 0.92) | 0.78 (0.66, 0.87) |
| Correctly classified proportion | 0.77 (0.67, 0.85) | 0.76 (0.66, 0.84) |
